# Three-dimensional modeling of in-ground cathodic protection systems with deforming anodes

**DOI:** 10.1038/s41598-021-81184-w

**Published:** 2021-01-21

**Authors:** Abraham Mansouri, Alreem Essa Binali, Najeeb Khan, Mehrooz Zamanzadeh, Peyman Taheri

**Affiliations:** 1 Department of Mechanical Engineering, DBM, HCT, Dubai, 15825 UAE; 2 Matergenics Inc, 100 Business Center Drive, Pittsburgh, PA 15205 USA

**Keywords:** Computational methods, Mechanical engineering

## Abstract

The design of sacrificial cathodic protection (CP) systems conventionally involves steady-state assumptions, which means design parameters are considered constant during the in-service life of CP systems. In contrast, it is evident by experimental observations (including field measurements) that cathodic protection is a transient process due to variations in electrolyte properties such as seasonal changes in electrical conductivity of soil, depletion of anodes, and formation of corrosion deposits on anode material surface, to name a few. The lack of practical time-dependent models on this critical issue is apparent in the literature; accordingly, in this study, a pseudo transient electrochemical model is adopted to highlight the transient behavior of cathodic protection systems and investigate key differences with steady-state behavior. For the sake of demonstration, the developed model is used to simulate the time-dependent performance of a sacrificial anode bed for cathodic protection of screw-pile foundations. The methodology proposed in this study can be used by corrosion engineers to improve and optimize the design of CP systems and numerically estimate the performance of sacrificial anodes and the level of protection over time.

## Introduction

Cathodic protection (CP) is proved to be an effective and cost-effective method for underground corrosion control, and it is increasingly used to protect steel pipelines and other below-grade assets against corrosion damage. Conventionally, the parameters involved in the design of CP systems are considered as constants. However, a relatively large safety factor is included in the design to compensate for the temporal variation of design parameters.

Cathodic protection design parameters can be divided into three groups: (a) Parameters that define the anode bed, (b) Parameters that define the structure to be protected (cathode), and (c) Parameters that define the electrolyte, i.e., a corrosive environment such as soil or water. In real-world applications, all mentioned design parameters could be considerably diverted from a fixed design value assumption. For example, in the case of a coated structure, the performance of the coating system changes with time as the coating material degrades. Similarly, performance parameters of anodes change as they corrode, and a passive layer is developed on their surface as they age^1–3^. Another significant transient effect is the seasonal variation of soil resistivity due to changes in water content and temperature^4^. It is evident that a structure may become under-protected due to these time-dependent effects.

Numerical analysis of cathodic protection systems can be divided into two categories: finite-element method (FEM), and boundary-element method (BEM). One of the earliest works on modeling the CP system was carried by Montoya *et al.*^5^ where a FEM model was developed to solve the Laplace equation in a two-dimensional rectangular domain. In Ref.^5^ parameters such as low-conductivity irregularities in the electrolyte, electrode distribution in the system, and different electrolyte resistivities were investigated. Liu and Kelly^6^ conducted an advanced review on the application of FEM modeling to localized corrosion. Parsa *et al.*^7^ employed a finite-element analysis for the evaluation of the design of cathodic protection systems in oil well castings. Their findings confirmed a strong correlation between soil conductivity and potential distribution. Santos *et al.*^8^ compared the method of fundamental solutions and direct boundary elements for the solution of Laplace׳s equation and analyzing the cathodic protection systems. Kim et al*.*^9,10^ developed a boundary-element numerical model to optimize the cathodic protection designs based on the anode lifetime provided and cathodic potential distribution. They experimentally showcased the corrosion at unprotected samples and made a comparison with protected ones. In another study, Marcassoli et al*.*^11^ adapted a finite-element model for cathodic protection simulation in subsea pipelines. Their model was based on a simplified two-dimensional domain with physical and electrochemical parameters, such as mud burial depth, sea depth, seawater and mud resistivity, and potential and current density distribution^11^. While the majority of studies in the literature carried out steady-state modeling of cathodic protection, Saeedikhani et al*.*^12,13^ published studies on time-dependent behavior of zinc deformation/consumption in galvanized steel using the moving boundary simulation method. Sun et al*.*^14^ adapted an Arbitrary Lagrangian–Eulerian (ALE) numerical model for the time-dependent evolution of crevice corrosion. The time-dependent deformation of the crevice geometry, due to metal dissolution, was shown via the ALE method^14^. These recent articles studied the corrosion-induced deformations, but their models were limited to two-dimensional domains.

It is understood by the authors that cathodic protection is a mature subject, and its applications and the underlying processes are well-documented in the literature. Nonetheless, the lack of a "transient three-dimensional" model is apparent in literature for real-world engineering applications. All published numerical studies, as referenced in "Electrode Deformation" section, are limited to planar two-dimensional geometries, including a galvanic coupling (single anode and cathode sites) with minimal geometry features. The proposed case in this manuscript is computationally expensive and requires several hours to complete anode deformation simulations; the addition of further details to the model, such as the formation of depositions on the anodes' surface, is identified as a computationally challenging task.

This study is a sequel to our previous research in which the authors developed a numerical model to solve three-dimensional potential and current distribution fields associated with galvanic CP systems. In previous works, we addressed some of the technical issues in the design and optimization of anode beds for cathodic protection of irregular geometries in steady-state conditions^15,16^. This paper presents an improved model with a focus on the transient nature of the corrosion process. As the first step, we have considered the effects of anodes deformation on the performance of CP systems along with a parametric study on soil resistivity. A three-dimensional ALE method, coupled with Faraday's laws of electrolysis, is used to calculate the rate and pattern for anodes depletion. The approach is showcased for cathodic protection of a steel screw-pile foundation in the soil environment.

## Anode bed design base on steady assumptions

For the sake of simplicity, a cathodic protection system for a single screw-pile is considered to demonstrate the modeling approach. This screw pile can be considered as an anchor-shaft foundation for a utility structure such as telecom or transmission structures. The screw-pile is made of structural steel (ASTM A2527), with a length of 9.7 m (32 ft) and an outer diameter of 32 cm (12.75 in). The external surface area of the screw-pile in contact with soil is 11 m^2^ (120 ft^2^).

**Figure 1:**
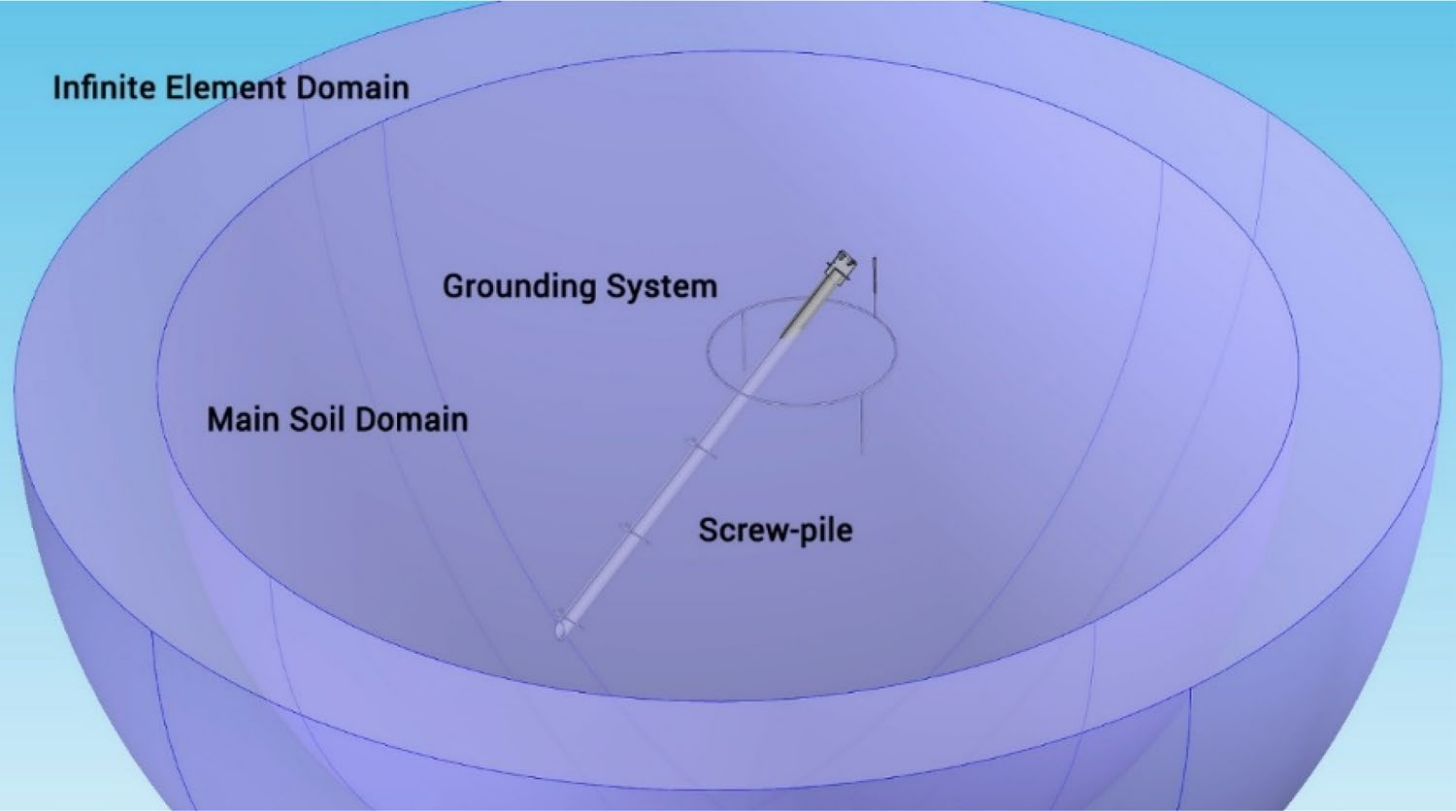
Three-dimensional geometry model for the considered screw-pile foundation.

As shown in Fig. 1, the screw-pile foundation includes a grounding system with three spikes, which are connected with a grounding loop. The components of the grounding system (the loop and spikes) are made of copper cables and rods with a diameter of 1 inch. The buried surface area of the grounding system is 1.3 m^2^ (14 ft^2^). A hemisphere of soil around the screw-pile is selected as the main electrolyte domain. Also, an infinite element domain with proper boundary conditions is considered around the main domain to take account of the effects of the infinite soil environment.

The native potential of the buried system is measured as − 700 mV-CSE, and resistivity tests indicate 4000 Ω-cm for the soil resistivity. The current requirement test with temporary drive-in anodes indicated that 110 mA is required for cathodic protection of buried components; this results in a current density of 10 mA/m^2^ (0.9 mA/ft^2^).

Based on measured soil resistivity value (4000 Ω-cm), high-potential magnesium anodes (Type M1, per ASTM B843) are selected^17^, and a nominal lifespan of 30 years is considered for the CP system. This preliminary information can be used to calculate the capacity of the CP system from the following formula:

1 $${Q}_{cp}={I}_{cp}\times {\text{Life}}$$

where ${Q}_{cp}$ is the capacity of the cathodic protection system (A-yr), ${I}_{cp}$ is the required CP current (A), and ${\text{Life}}$ is its nominal lifespan in years. Based on Eq. (1), the required capacity of the CP system is calculated as 3.3 A-yr.

The minimum required mass of magnesium anode for the calculated CP capacity can be obtained from the following equation:

2 $${m}_{a}={Q}_{cp}/\left({Q}_{a}\times E\times U\right)$$

where ${m}_{a}$ is the minimum required mass of anode material (kg), and ${Q}_{a}$ is the theoretical capacity of anode material, which is 0.251 A-yr/kg for selected magnesium anodes^18,19^. The parameter $E$ is the current efficiency of anode material and is 50% for high-potential magnesium anodes. Parameter $U$ is a utilization factor for magnesium anodes, as is considered as 85%^4,19^. From the above equation, the minimum anode mass for cathodic protection can be calculated as 30.9 kg (68.1 lb). In conventional CP design, it is a common practice to consider a safety factor to increase the mass of anodes and take account for transient effects such as anodes passivation. No safety factor is considered here.

**Figure 2:**
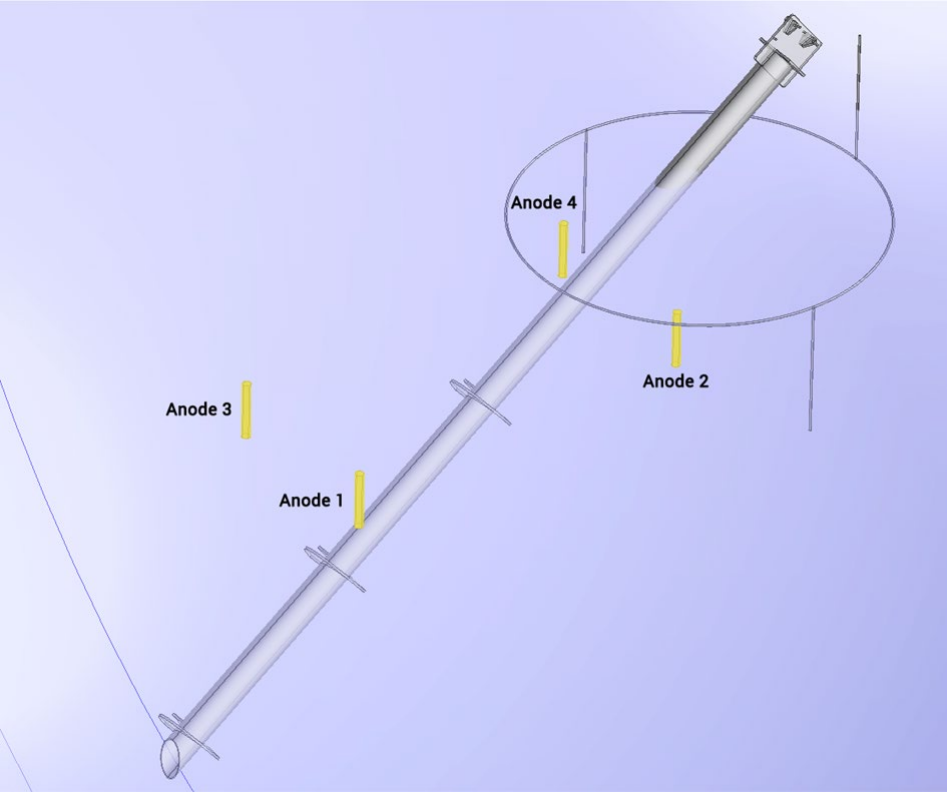
An anode bed with 4 × 17 lb high-potential magnesium anodes is considered for cathodic protection.

Based on the above calculations, an anode bed with 4 × 7.7 kg (17 lb) anodes is considered. As shown in Fig. 2, anodes are placed vertically around the anchor shaft. The depth of anodes is 1.5–2 m (5–6.5 ft.), and their lateral distance from the screw-pile axis is 1 m (3.3 ft.). Anodes are cylindrical with 9.1 cm diameter and 65 cm length.

In this study, anodes are considered in a fixed location, and numerical simulations are performed to investigate the effect of anode deformation on the performance of the CP system. Numerical simulations can also be performed for different configurations of anodes around the screw-pile to find the best anode arrangement; however, anode bed optimization is not considered in this study.

### Numerical model

### Electrolytic current

The mass transport in the soil (electrolyte) between anodes and the screw-pile (cathode) is governed by:

3 $$\frac{\partial {c}_{i}}{\partial t}+\nabla \cdot {\mathbf{N}}_{i}=0$$

where ${c}_{i}$ is the concentration (mol/cm^3^) of species $i$, $t$ is time (s) and ${\mathbf{N}}_{i}$ is the mass flux vector (mol/cm^2^ s) of species $i$ based on the Nernst-Planck equation^20^:

4 $${\mathbf{N}}_{i}={c}_{i}\mathbf{V}-{z}_{i}{u}_{i}F{c}_{i}\nabla \varphi -{D}_{i}\nabla {c}_{i}$$

The terms in Eq. (4) respectively correspond to convection, migration, and diffusion transport of ions in the electrolyte, which all together contribute to the electrolytic current. $\mathbf{V}$ is the bulk electrolyte velocity (cm/s) vector, ${z}_{i}$ is charge number of species $i$, ${u}_{i}$ is the mobility coefficient (mol cm^2^/J s) of species $i$, $F$ is Faraday's constant (96,487 C/mol), $\varphi$ is the electric potential (V), and ${D}_{i}$ is the diffusion coefficient (cm^2^/s) of species $i$. The mobility and diffusion coefficients for species $i$ are related by the Nernst–Einstein equation ${u}_{i}={D}_{i}/(RT)$, $R$ is the universal gas constant (8.3145 J/mol K), and $T$ is the absolute temperature (K).

The electrolytic current density vector $\mathbf{i}$ (A/cm^2^) is the summation of iconic current for all species involved and can be calculated from Eq. (4) as:

5 $$\mathbf{i}=F{\sum }_{i}{z}_{i}{\mathbf{N}}_{{\varvec{i}}}$$

By substituting ${\mathbf{N}}_{i}$ from Eq. (4) into Eq. (5), the continuity equation $\nabla\cdot \mathbf{i}=0$ can be simplified as Eq. (6) below by assuming electroneutrality condition and neglecting convection and diffusion contributions in the electrolytic current.

6 $${-F}^{2} \nabla \cdot \nabla \varphi \sum_{i}{z}_{i}^{2}{u}_{i}{c}_{i}=0$$

Equation (6) can be recast into the well-known Laplace equation for electrical potential:

7 $$-\sigma {\nabla }^{2}\varphi =0$$

with,

8 $$\sigma ={F}^{2} {\sum }_{i}{z}_{i}^{2}{u}_{i}{c}_{i}$$

where $\sigma$ (S/cm) is the conductivity of the electrolyte. The current density in the electrolyte due to charge transport can be written in the ohmic form^20^:

9 $$\mathbf{i}=\sigma \nabla \varphi$$

#### Boundary conditions

Tafel equations are used as boundary conditions to define electrochemical kinetics on anodic and cathodic surfaces and to relate current densities at electrode–electrolyte interfaces to electrode and electrolyte potentials. Tafel expressions in the following form are used^20^:

10 $${i}_{a}\left(\varphi \right)={i}_{0,a}\times {10}^{\frac{\eta \left(\varphi \right)}{{A}_{a}}}\,\,\,\mathrm{ and }\,\,\,{i}_{c}\left(\varphi \right)={i}_{0,c}\times {10}^{\frac{\eta \left(\varphi \right)}{{A}_{c}}}$$

where $i\left(\varphi \right)$ is the current density at the electrode–electrolyte interface (A/cm^2^), ${i}_{0}$ is the exchange current density (A/cm^2^), $A$ is the Tafel slope (V), and $\eta$ is the over-potential (V):

11 $${\eta }_{a}={\varphi }_{a}-{\varphi }_{e}-{E}_{\mathrm{eq},a}\,\,\,\mathrm{ and }\,\,\ {\eta }_{c}={\varphi }_{c}-{\varphi }_{e}-{E}_{\mathrm{eq},c}$$

The subscript $a$, $c$ , and $e$ correspond to the anode, cathode, and electrolyte. The parameter ${E}_{\mathrm{eq}}$ is the equilibrium potential (V) of electrodes.

The parameters in Eqs. (10) and (11) define the kinetics of electrochemical processes at the surface of anode and cathode, and their values depend on electrode material and electrolyte properties.

Potentiostatic tests are required to evaluate Tafel slopes. In soil applications, Tafel parameters vary with time as soil properties change seasonally. Accordingly, it is a common practice to evaluate the Tafel slope for saturated soil samples, which represent the most corrosive state of the soil service environment and implement the values in electrochemical simulations. Indicative values for the Tafel parameters used in this study are listed in Table 1. In simulations, an average equilibrium potential of − 700 mV-CSE is assumed for the steel screw pile and -350 mV-CSE for grounding copper. The equilibrium potential of anodes in their backfill material is almost constant and around − 1750 mV-CSE.

**Table 1:** Tafel parameters for anode and cathode materials in contact with the soil environment.

${{\varvec{A}}}_{{\varvec{a}}}$(V-SCE)	${{\varvec{A}}}_{{\varvec{c}}}$(V-CSE)	${{\varvec{i}}}_{0,{\varvec{a}}}$(A/m^2^)	${{\varvec{i}}}_{0,{\varvec{c}}}$(A/m^2^)	${{\varvec{E}}}_{\mathbf{e}\mathbf{q},{\varvec{a}}}$(V-CSE)	${{\varvec{E}}}_{\mathbf{e}\mathbf{q},{\varvec{c}}}$(V-CSE)
0.050	− 0.160 (steel) − 0.100 (copper)	0.100	0.001 (steel) 0.001 (copper)	− 1.750	− 0.700 (steel) − 0.350 (copper)

#### Electrode deformation

The challenging part of this study was the numerical implementation of three-dimensional anode deformation in multiple anode sites, as shown in Fig. 2. The corrosion morphology of anodes surface includes two steps; first, dissolution of magnesium on the anode surface and release of magnesium ions into the liquid phase of soil (electrolyte); secondly, the reaction of magnesium ions with water molecules or other anions in the electrolyte that results in formation and deposition of the corrosion product on the anode surface. It is well-known that the deposition of corrosion products on the magnesium surface changes its surface and can significantly affect the electrochemical behavior at the metal-electrolyte interface^21^. Depending on the species present in the electrolyte, corrosion products can be metal-oxide semiconductors^22–24^, insulating films of metal salts^25,26^, porous layers^27,28^, or dense layers^29^. When corrosion products form impervious and tenacious layers on the anode surface, the corrosion rate will be considerably reduced^1–3^. On the contrary, when corrosion products are porous, not only they do not have a protective effect^30–32^ but they can also result in accelerated localized corrosion such as pitting, crevice corrosion, and microbiologically-induced corrosion on the anode surface^33–35^.

An Arbitrary Lagrangian–Eulerian (ALE) numerical technique is employed in this study, which allows boundary movement on the anodes only due to dissolution of magnesium, $\mathrm{Mg}\to {\mathrm{Mg}}^{2+}+2{e}^{-}$. In this study, deposition of corrosion products and formation of a deposited film on anodes' surface is not implemented as a result of an extreme computation demand. This is due to the complex nature of chemical and electrochemical reactions, the three-dimensional geometry of anodes^21^. Published studies^14,21^ that include deposition of corrosion products on anode sites are limited to planar two-dimensional geometries with minimal geometry features that are computationally manageable.

Tetrahedral cells in the Cartesian coordinates are used to generate the computation grid in the electrolyte domain. For a three-dimensional model with free displacement in the transient case, the displacement of the computational grid is governed by the following equations:

12a $$\frac{\partial }{\partial {X}^{2}}\frac{\partial x}{\partial t}+\frac{\partial }{\partial {Y}^{2}}\frac{\partial x}{\partial t}+\frac{\partial }{\partial {Z}^{2}}\frac{\partial x}{\partial t}=0$$

12b $$\frac{\partial }{\partial {X}^{2}}\frac{\partial y}{\partial t}+\frac{\partial }{\partial {Y}^{2}}\frac{\partial \mathrm{y}}{\partial t}+\frac{\partial }{\partial {Z}^{2}}\frac{\partial \mathrm{y}}{\partial t}=0$$

12c $$\frac{\partial }{\partial {X}^{2}}\frac{\partial z}{\partial t}+\frac{\partial }{\partial {Y}^{2}}\frac{\partial \mathrm{z}}{\partial t}+\frac{\partial }{\partial {Z}^{2}}\frac{\partial z}{\partial t}=0$$

where $\mathbf{x}=(x,y,z)$ and $\mathbf{X}=(X,Y,Z)$ correspond to the fixed spatial frame and the moving material frame, respectively.

Anode deformation is physics-induced and must be related to the local rate of magnesium dissolution at the anode-electrolyte interface. These reactions cause anode domains to shrink at a local velocity that directly depends on current density distribution on their surface, as presented in Eq. (10). This velocity can be calculated from Faraday's law of electrolysis:

13 $$\mathbf{n}\cdot \mathbf{v}=\frac{{i}_{a}\left(\varphi \right) {M}_{\mathrm{Mg}}}{{z}_{\mathrm{Mg}} F {\rho }_{\mathrm{Mg}}}$$

where $\mathbf{n}$ is the surface normal vector $\mathbf{v}$ is the boundary movement velocity vector (cm/s), ${M}_{\mathrm{Mg}}$ is the mean molecular mass for anode material (25 g/mol for the magnesium alloy), ${z}_{\mathrm{Mg}}$ is the charge number for anode material (2 for magnesium), $F$ is the Faraday's constant (96,487 C/mol), and ${\rho }_{\mathrm{Mg}}$ is the density of anode material (1.82 g/cm^3^ for the magnesium alloy).

## Results and discussions

A commercial finite-element PDE solver is used to solve the governing equations, obtain three-dimensional distributions of potential and ionic current, investigate the pattern of anode deformation and its effect on the cathodic protection performance.

**Figure 3:**
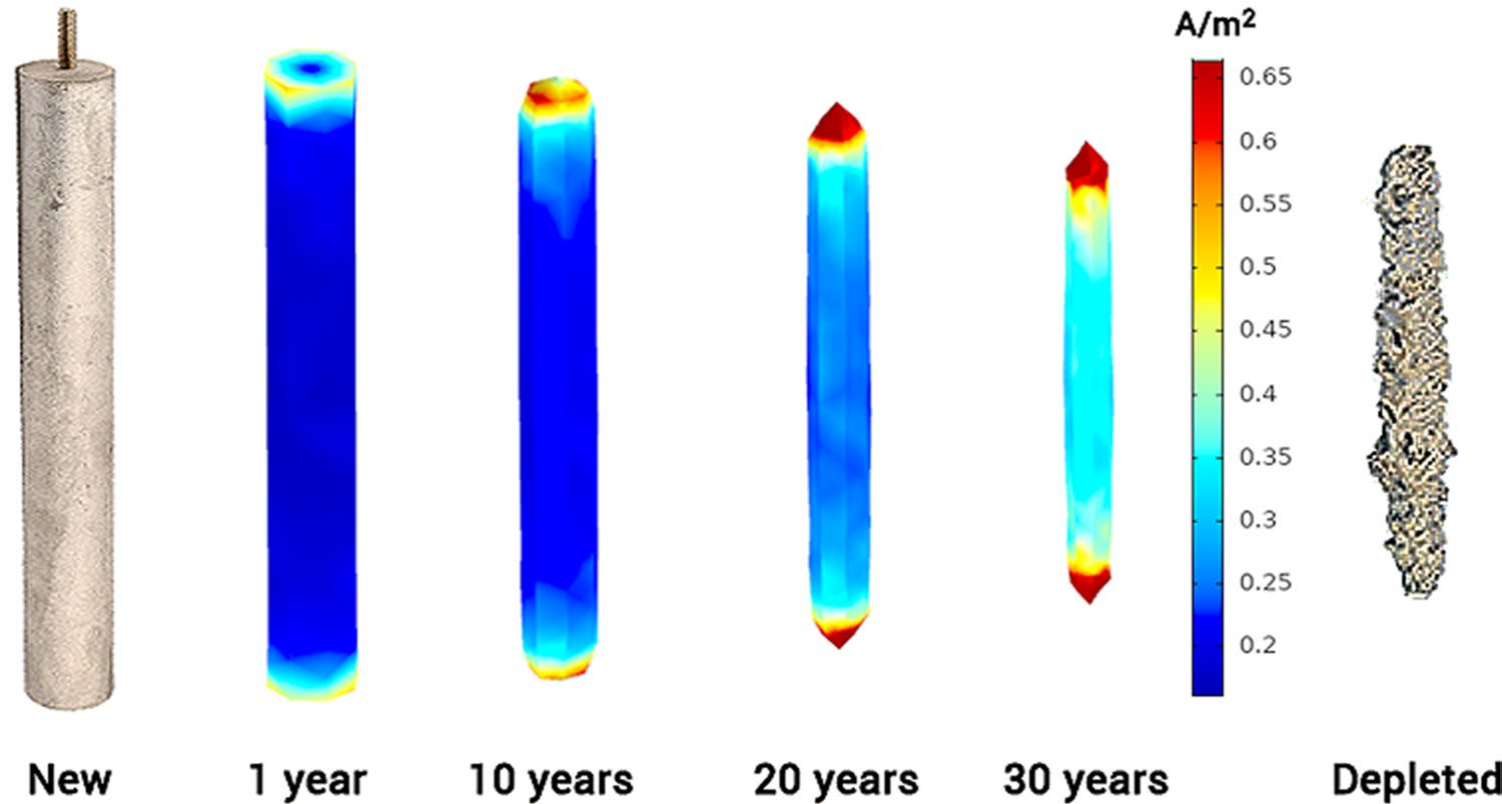
Current density (A/m^2^) distribution and anode depletion pattern during in-service life of 30 years, with a soil resistivity of 4000 Ω-cm.

In Fig. 3, the distribution of current density on one of the anodes is shown for different time stamps during its in-service life. The simulation results confirm that areas with maximum current discharge are the top and bottom edges of anodes. Accordingly, maximum material depletion occurs in these areas. This pattern, known as "anode end effect", is observed in real-world observations when aged anodes are taken out of service—a sample photo of a depleted anode is shown in Fig. 3. It is important to mention that formation of deposits on anode surface is not considered in this study; therefore, variations in corrosion morphology are expected between simulations and actual cases.

**Figure 4:**
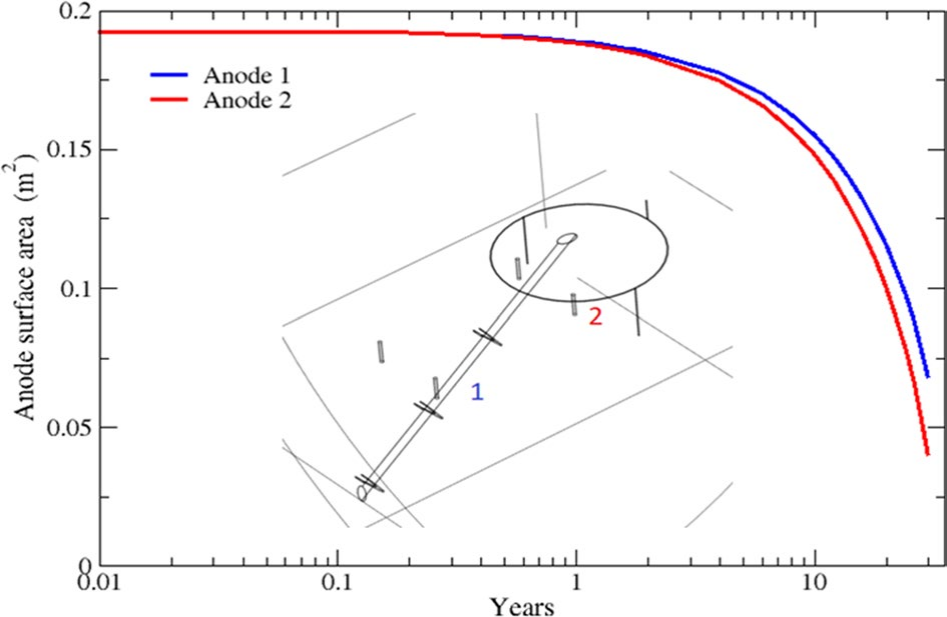
Comparison of surface area reduction for two anodes due to material depletion. Change in the anodes surface area is plotted over 30 years for soil resistivity of 4,000 Ω-cm.

Anodes deletion in terms of reduction in their surface area is plotted over their in-service life (30 years), as shown in Fig. 4. The results are plotted for two anodes with different locations with respect to the pile. The plots confirm that the anode closer to the grounding system (i.e., anode 2) is depleted at a slightly higher rate owing to a higher discharge current.

As shown in Eq. (9), both potential ($\varphi$) and current density ($\mathbf{i}$) fields depend on the soil resistivity ($\sigma$) value. Since Tafel parameters are functions of potential, they are also dependent on soil resistivity. This means that soil resistivity in this model is the most influential parameter that can significantly affect the performance of the cathodic protection system. Accordingly, a parametric study is conducted to investigate the sensitivity of the cathodic protection system to variations in soil resistivity.

**Figure 5:**
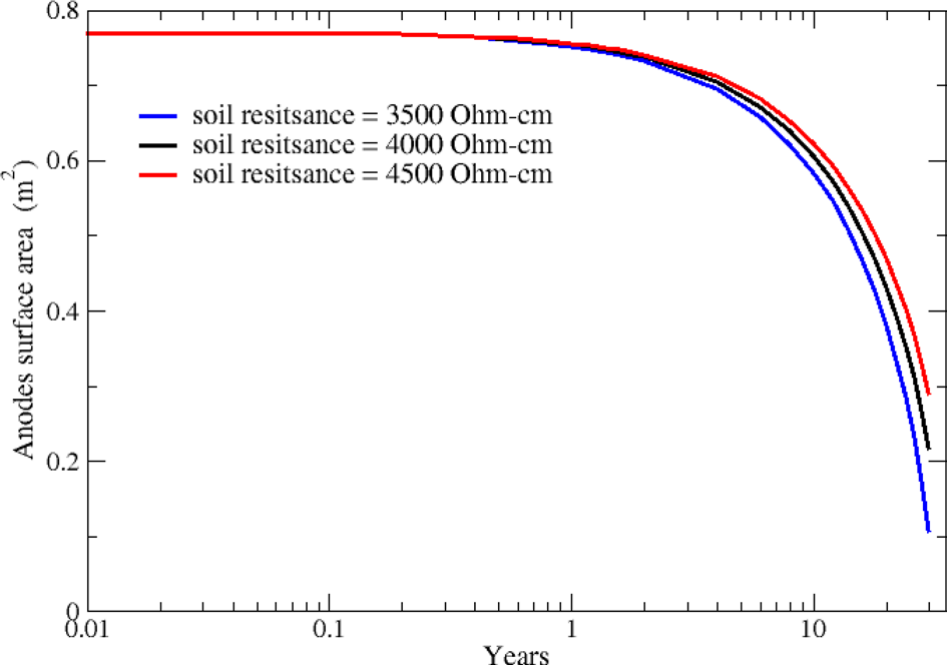
Surface area reduction for all anodes in soils with different resistivity values.

In Fig. 5, surface area reduction for all four anodes is plotted for soil resistivity values of 3500, 4000, and 4500 Ω-cm. As expected, the rate of anode shrinking is in inverse relation with soil resistivity, i.e., in less resistive soils, more current can be discharged from anodes; thus, anodes deplete faster. The surface area of corroding anodes decreases by as much as 65% over 30 years. Finite element simulations beyond the 3500 Ω-cm soil resistivity did not converge over 30 years due to excessive corrosion in the anodes.

**Figure 6:**
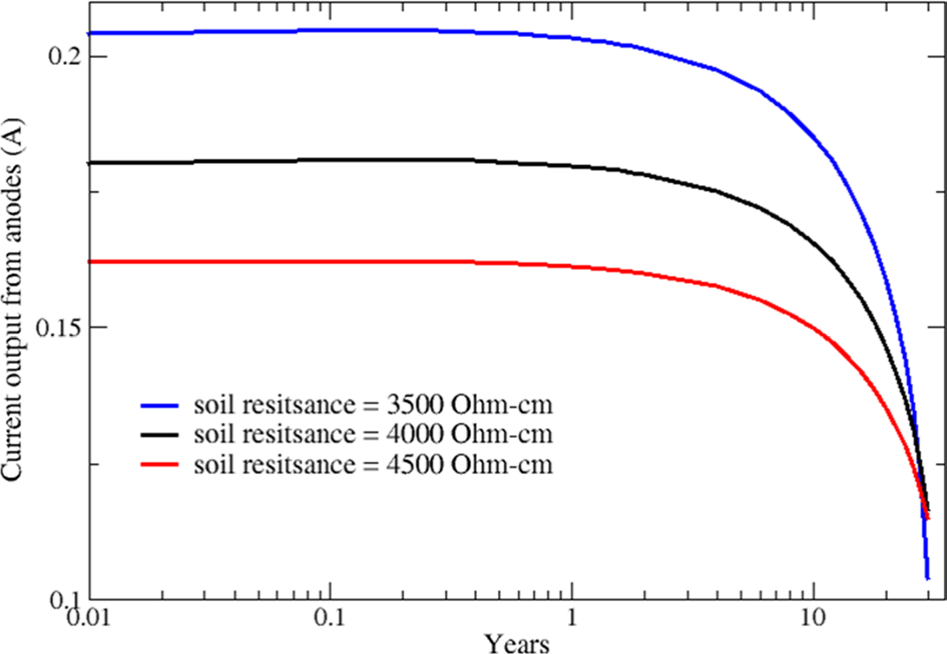
Discharge current from all anodes in soils with different resistivity values.

In order to exhibit how anodes discharge current changes with soil resistivity, the total discharge current of anodes for various soils resistivities are plotted in Fig. 6. The maximum current is observed for the most conductive (least resistive) soil, which results in the maximum depletion. As shown in Fig. 6, the trend for current output changes at anodes' end-of-life. This is because anodes in high resistivity soil deplete slower and have a larger surface area compared to anodes in low resistivity soils.

**Figure 7:**
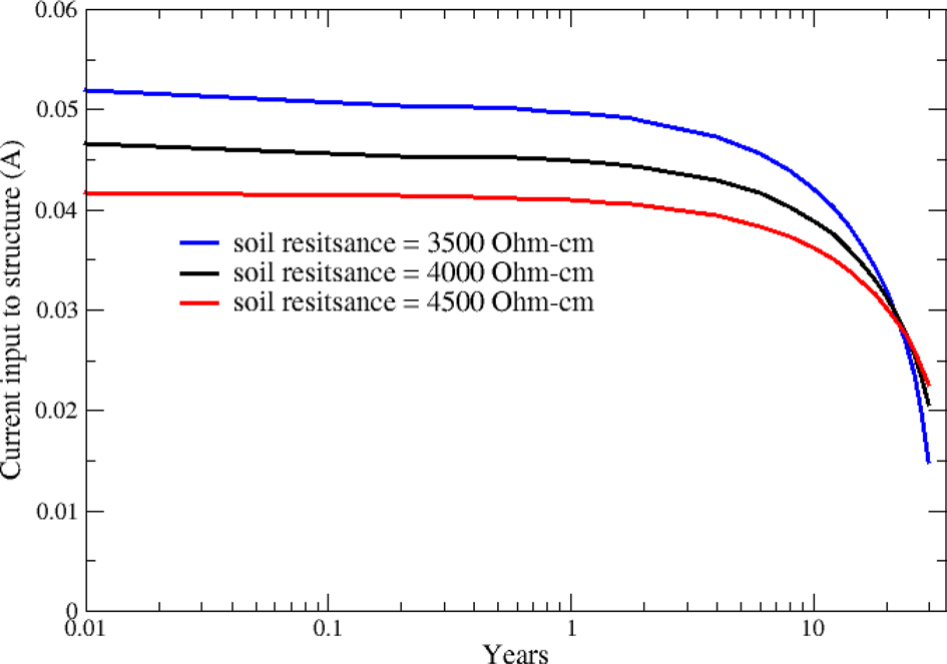
Variation of cathodic protection current at the screw pile over 30 years.

**Figure 8:**
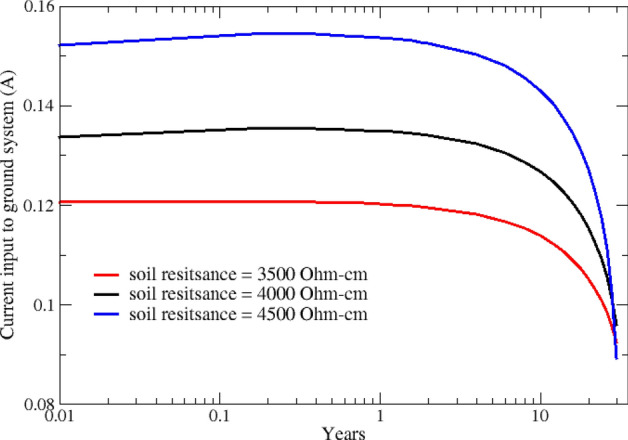
Variation of cathodic protection current at the grounding system over 30 years.

In the following, the distribution of the cathodic protection current between the steel structure (screw pile) and the copper grounding system is investigated. The total CP current discharged from anodes, shown in Fig. 6, is split between the screw pile and the grounding system. Simulation results show that a significant portion of the CP current, around 70%, is received by the grounding system, and only 30% is used for cathodic protection of the pile. Figures 7 and 8 show how CP current is shared between different components and their variation over 30 years for various soil resistivities.

After excluding the copper grounding system from simulations, we observed improved cathodic polarization on the pile surface, especially in shallow burials. Without the grounding system, the lifespan of the CP system extends to as many as 44 years. The leak of CP current to grounding systems is a well-known issue, especially for structures with extended grounding systems, such as structures inside substations. To address this issue, modification of the grounding system and/or installation of high-capacity anode beds are recommended.

**Figure 9:**
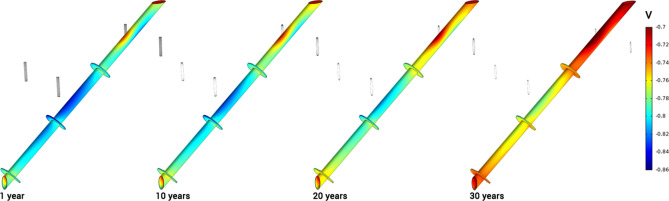
Variation of potential (V-CSE) distribution on the pile is shown for different years after CP system commissioning.

In Fig. 9, three-dimensional distributions of potential on the pile surface are shown for different years after CP system commissioning. Simulation results confirm a significant decrease in cathodic polarization as the anodes deplete. Based on the NACE SP0169 standard^36^, potential values can be used to quantitatively assess the level of cathodic protection. According to the 100 mV cathodic polarization criterion, areas showing − 0.8 V-CSE or higher are fully protected. Considering the NACE's protection criteria, the middle and bottom sections of the pile exhibit higher protection levels compared to its top section. This is because, at the top section, most of the protection current is consumed by the grounding system, as discussed above, and shown in Figs. 6, 7 and 8.

**Figure 10:**
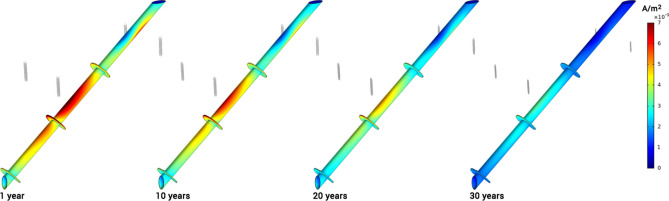
Variation of current density (A/m^2^) distribution on the pile is shown for different years after CP system commissioning.

Figure 10 shows the three-dimensional current density distribution on the pile surface for different years of in-service life. As expected, the pattern for the current distribution is similar to the potential for the same reasons.

**Figure 11:**
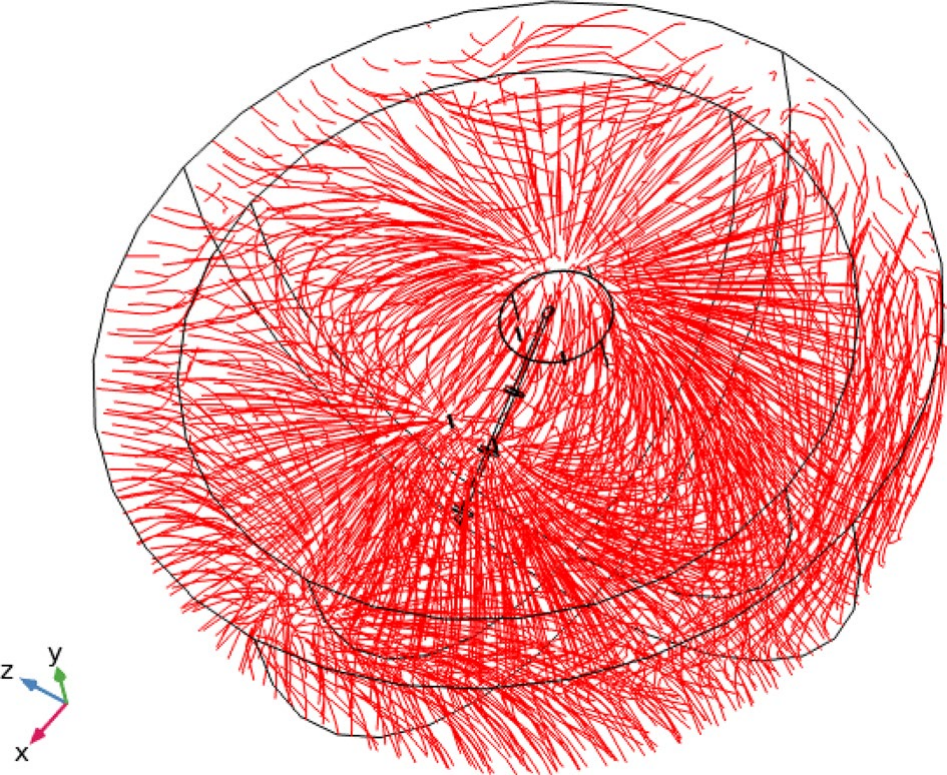
Streamlines of cathodic protection current in the soil after 30 years.

In Fig. 11, the streamlines of the current density vector corresponding to the cathodic protection current in the soil are shown for the anode arrangement. The streamlines only qualitatively show the formation of the current field, and their change with anode deformation is negligible. Our simulation confirms that the current distribution field does not change qualitatively during the lifespan of the CP system.

## Conclusion

A pseudo-transient numerical approach is presented to study the performance of sacrificial cathodic protection systems with high-potential magnesium anodes for corrosion protection of underground steel assets. Finite-element calculations are performed to simulate three-dimensional distributions of potential and current density in the soil environment and on the surface of the buried steel structure. Depletion rate and pattern of anode deformation are predicted and compared to real-world observations. For the considered case, it is shown that after 30 years of in-service life, the surface area of the sacrificial anode is decreased by 65%, and as a result, the level of protection was drastically affected. The detailed simulations enable engineers and corrosion professionals to estimate the effects of anodes deformation and electrochemical parameters on the performance of CP systems and to predict the efficient in-service life of anode beds in order to achieve the desired level of cathodic protection. In addition, the presented approach is able to capture the interaction between individual anodes in the anode bed resulting in different anode depletion rates.

Finally, in the presented study, the effect of CP current leak to foreign/auxiliary structures (such as grounding systems) is investigated. The findings of this study can be used as guidelines for corrosion engineers to optimize the CP systems for complex design cases and monitor the performance of anodes and level of protection over time.
